# Complete Genome Sequence of *Escherichia coli* BW25113

**DOI:** 10.1128/genomeA.01038-14

**Published:** 2014-10-16

**Authors:** Frédéric Grenier, Dominick Matteau, Vincent Baby, Sébastien Rodrigue

**Affiliations:** Département de Biologie, Université de Sherbrooke, Sherbrooke, Canada

## Abstract

*Escherichia coli* BW25113 is the parent strain of the Keio collection comprising nearly 4,000 single-gene deletion mutants. We report the complete 4,631,469-bp genome sequence of this strain and the key variations from the type strain *E. coli* MG1655.

## GENOME ANNOUNCEMENT

*Escherichia coli* BW25113 is a common laboratory strain that was created in the laboratory of Barry L. Wanner and was utilized in a method taking advantage of the bacteriophage lambda red recombination system to perform gene disruptions with double-stranded PCR products (1). *E. coli* BW25113 later became the parent strain for the Keio collection, a major resource consisting of approximately 4,000 single-gene deletion mutants (2, 3). The strain and its derivatives are being used in countless laboratories for a variety of studies, including systematic phenotypic surveys (4) and synthetic biology efforts (5–7). Despite this, the complete genome sequence of this strain surprisingly remained unavailable for the scientific community.

*E. coli* BW25113 was obtained from the Coli Genetic Stock Center (CGSC) (strain 7636). An Illumina library was prepared from size-selected DNA fragments of approximately 450 to 550 bp and sequenced with paired-end reads of 300 bp on a MiSeq instrument to assemble longer composite reads covering the entire insert (8). All sequences were *de novo* and reference assembled using the Roche gsAssembler version 2.6. The assemblies were merged and manually inspected before manual finishing with Sanger sequencing reads obtained from PCR products. The resulting circular chromosome (of 4,631,469 bp) was annotated by comparison with *E. coli* MG1655 (RefSeq accession no. NC_000913.3) using RATT (9) and manual curation. The key differences between the two organisms were accounted for in the genotype of *E. coli* BW25113 [Δ(*araD-araB*)*567* Δ(*rhaD-rhaB*)*568 ΔlacZ4787* (::rrnB-3) *hsdR514 rph-1*], with the deletion of *araBAD* and *rhaDAB* and the replacement of a section of *lacZ* with four tandem *rrnB* terminators as well as a frameshift mutation in *hsdR* resulting in a premature translation stop codon. As noted by others (3), we observed that the strain contains the *lacI*^*+*^ allele and not *lacI*^*q*^ as initially reported (1, 10). The genome sequence also confirmed the presence of the *rph-1* allele and revealed 20 substitutions as well as 11 indels (see http://bioinfo.ccs.usherbrooke.ca/BW25113.html for a complete list).

### Nucleotide sequence accession number.

The complete genome sequence of *Escherichia coli* BW25113 was deposited in GenBank under accession number CP009273.
